# Supplementary material for: Post COVID-19 conditions in an Australian pediatric cohort, 3 months following a Delta outbreak
Source: Pediatr Res. 2024 Aug 30;97(5):1668–75. doi: 10.1038/s41390-024-03492-x (PMC12119362; doi:10.1038/s41390-024-03492-x)
Supplement: Supplementary file 1 — Supplementary Figures [file 41390_2024_3492_MOESM1_ESM.pdf]

Supplementary Figures

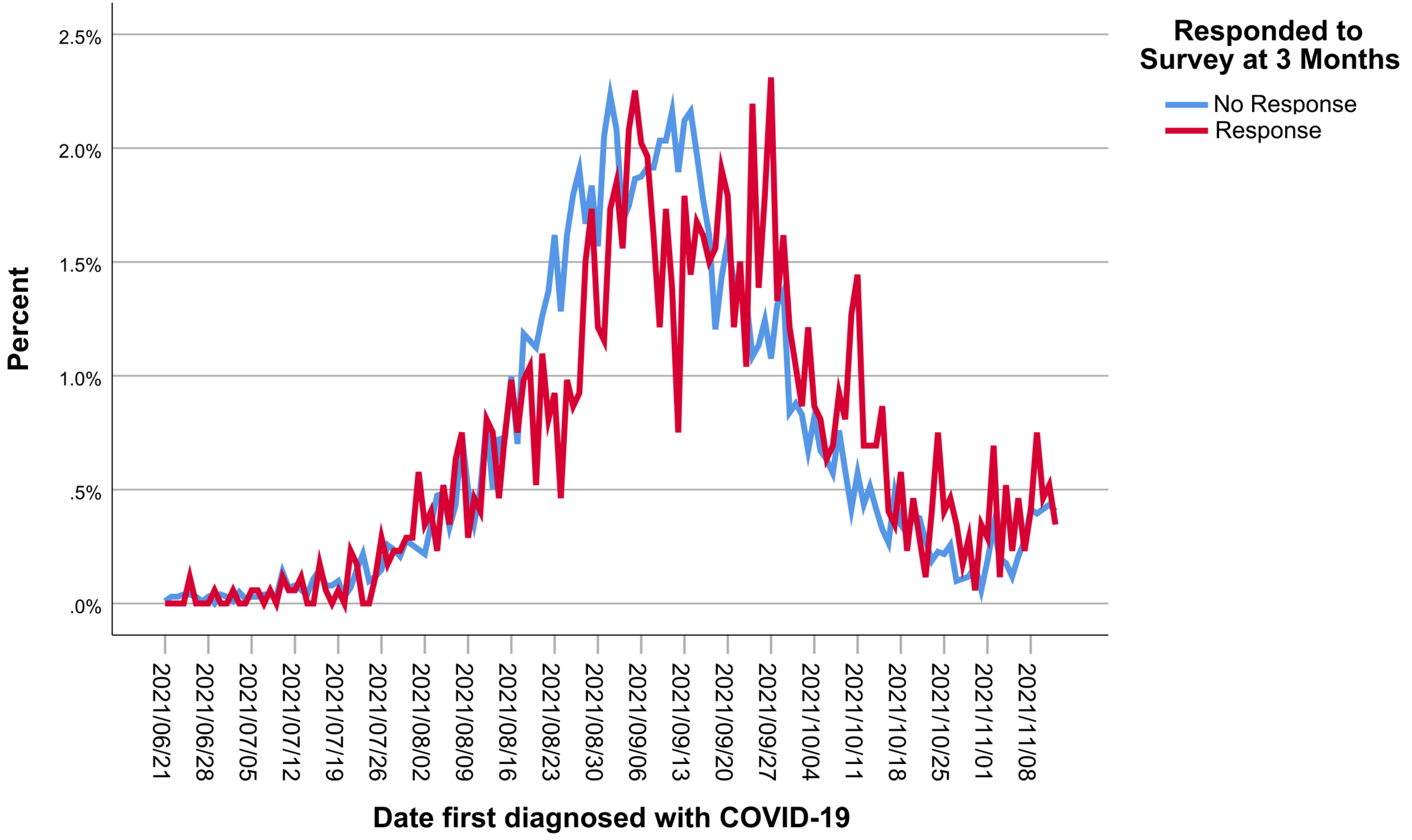

Supplementary Figure 1: Daily percentage of responder and non-responder cohorts to follow-up questionnaire by date of SARS-CoV-2 infection week across the 2021 Delta variant of concern (VoC) outbreak in Metropolitan Sydney.

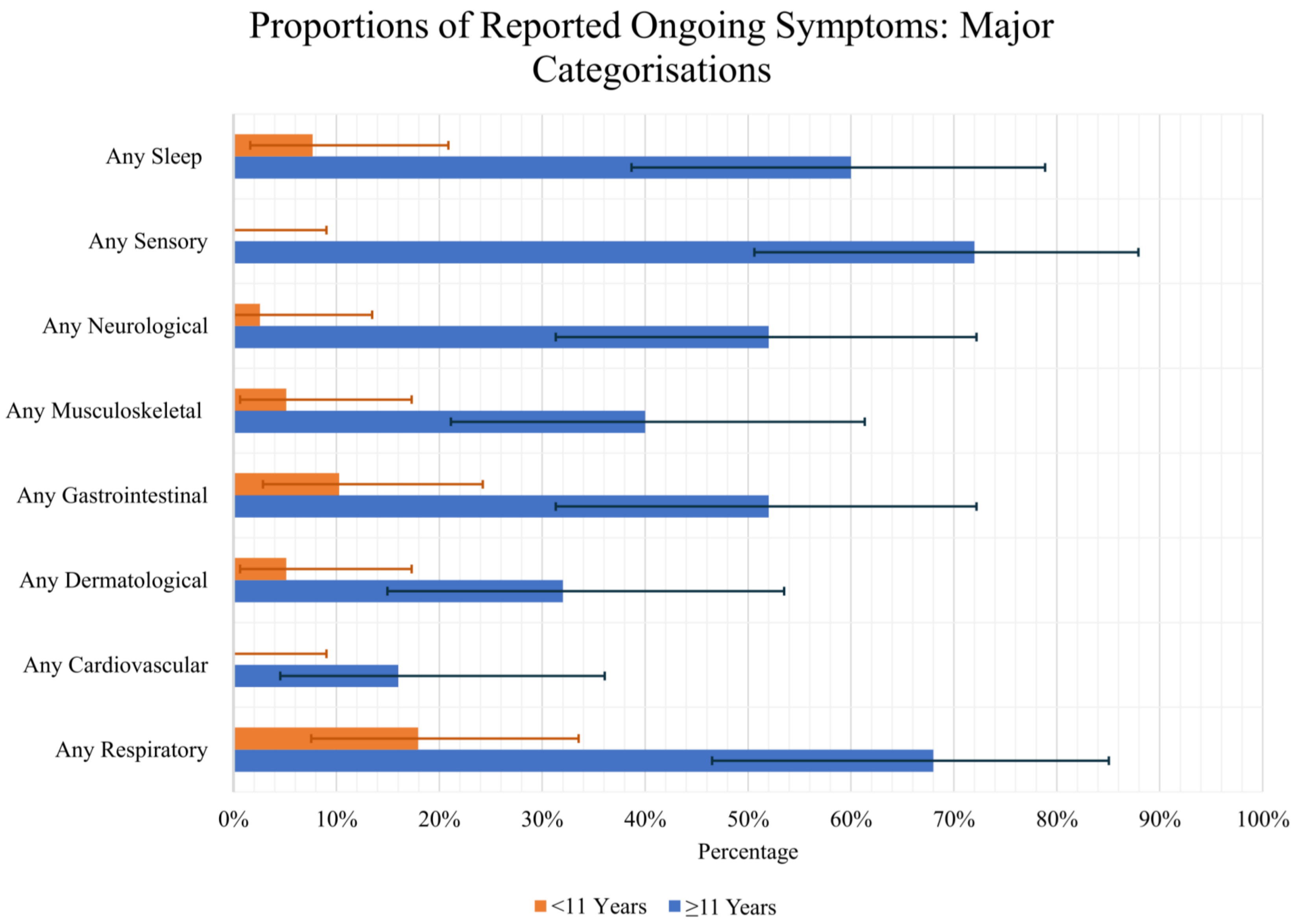

Supplementary Figure 2: Bar graph of self-reported persistent symptom categories (percentage and 95% confidence intervals) using ISARIC symptom questionnaire symptom list stratified by age; younger children aged <11y, older children aged ≥11y (n=64).
