# Supplementary material for: Post COVID-19 conditions in an Australian pediatric cohort, 3 months following a Delta outbreak
Source: Pediatr Res. 2024 Aug 30;97(5):1668–75. doi: 10.1038/s41390-024-03492-x (PMC12119362; doi:10.1038/s41390-024-03492-x)
Supplement: Supplementary file 2 — Supplementary Methods [file 41390_2024_3492_MOESM2_ESM.pdf]

Supplementary Methods:

Box 1: Consensus Definition of Long COVID in Children.[9]

> A condition in which a child or young person has symptoms (at least one of which is a physical symptom) that:
>
> - Have continued or developed after a diagnosis of COVID-19 (confirmed with one or more positive COVID tests)
> - Impact their physical, mental or social wellbeing
> - Are interfering with some aspect of daily living (e.gg, school, work, home or relationships) and
> - persist for a minimum duration of 12 weeks after initial testing for COVID-19 (even if symptoms have waxed and waned over that period)

Outcome measures

Clinical concern and subsequent review were determined by responses to ‘flagging’ questions outlined in Table 1. Early in the follow-up process, we recognized that more than half of the flagged responses were for raised SDQ scores only. Following direct contact with the first 20 of such cases by a Child and Adolescent Psychiatrist, it was determined that the SDQ identified pre-existing emotional and behavioral issues rather than screening for new symptoms of concern that may be associated with Long COVID.

Procedures

The REDCap questionnaire sent to families following their child’s SARS-CoV-2 infection was hosted on the Sydney Local Health District (SLHD) server. Contact details (email, mobile phone) were bulk-uploaded onto the SLHD server following registration in VK-CORT.

Data collation and analysis

Duplicate questionnaire responses (n=5) were identified and manually checked for discrepancies (PB, RB). Responses were retained for analyses where one questionnaire was more complete (n=4). In the instance both responses were completed with minor discrepancies, the response most proximal to illness was retained for analyses.

REDCap questionnaire data, medical record data (EMR extraction of VK-CORT records), and clinical review data were merged using medical record number (MRN). Duplicates in EMR extraction were identified via MRN, name, and date of birth, and removed prior to merging.

Children within the post-viral fatigue and persistent symptoms (multiorgan) clinical sub-groups were considered to be potentially consistent with the UK consensus case definition for ‘Long COVID’ (Box 1) if they had:

1) Persistence of symptoms validated by a reviewing clinician, and
2) Functional impact due to their persistent symptoms (inability to participate in usual activities and/or non-attendance at school or early childhood education centre, excluding that because of public health orders)
