# Supplementary material for: Post COVID-19 conditions in an Australian pediatric cohort, 3 months following a Delta outbreak
Source: Pediatr Res. 2024 Aug 30;97(5):1668–75. doi: 10.1038/s41390-024-03492-x (PMC12119362; doi:10.1038/s41390-024-03492-x)
Supplement: Supplementary file 3 — Supplementary Tables [file 41390_2024_3492_MOESM3_ESM.pdf]

Table 1: Key results for pre-determined 'flagging' questions from questionnaire responses.

**Key question (response result)**	**n/N**	**% (95% CI)**
Is [your child] interacting as per their usual baseline (i.e. before COVID)? (NO)	104/1728	6·0 (4·9-7·3)
Does [your child] have ongoing symptoms that concern you related to their COVID-19 illness? (YES)	106/1731	6·1 (5·0-7·4)
Are [your child's] COVID-19 related symptoms stopping them from doing their usual activities? (YES)	52/1730	3·0 (2·3-3·9)
Do you think you need additional help for [your child] to recover from COVID-19?	30/1730	1·7 (1·2-2·5)
Any screening Q positive	129/1731	7·5 (6·3-8·8)
Additional 'flagging questions'		
ISARIC How much do you agree with the statement, "[My child] has fully recovered from COVID-19"? *(0-10 scale with 0=strongly disagree; result <2)*	9/1610	0·6 (0·3-1·1)
Pediatric Dyspnea Scale How much difficulty has [your child] had breathing in the last 7 days? (>='some')	30/1615	1·9 (1·3-2·6)
WHO DAS How do you rate [your child's] overall health in the past 30 days? (<=bad)	8/1576	0·5 (0·2-1·0)
WHO DAS Q6 Days absent from school/ preschool/ daycare in past 30 (>9)	33/1380	2·4 (1·7-3·3)
Has [your child] been readmitted to hospital after first acute covid-19 illness	15/1555	1·0 (0·5-1·6)
ISARIC symptom list-Greater than 3 symptoms	28/64	43·8 (31·4-56·4)
EQ-5D-Y Q4 Having Pain or Discomfort (a Lot)	2/657	0·3 (0·0-1·1)
Age 12+ K6 Q4 About how often has [your child] felt hopeless (most of the time or all the time)	6/408	1·5 (0·5-3·2)
Age 12+ K6 total score greater than 18	11/410	2·7 (1·4-4·8)
Age 4-12 SDQ Q26 Difficulties in emotions, concentration, behaviour or being able to get on with other people (severe)	10/687	1·5 (0·7-2·7)
Age 4-12 SDQ flag^	301/687	43·8 (39·0-49·1)
Any additional flagging q (excluding SDQ flag)	74/1731	4·3 (3·4-5·3)
Total flagged for clinical review (excluding SDQ flag)	203/1731	11·7 (10·3-13·3)

Abbreviations: ISARIC, International Severe Acute Respiratory and emerging Infection Consortium; WHO-DAS World Health Organization Disability Assessment Schedule, K6 Kessler 6 item distress scale, SDQ = Strengths and Difficulties Questionnaire.

^ Strengths and Difficulties Questionnaire flag included any of, SDQ total difficulties scale or subscales in "borderline" or "abnormal" range from original 2-band categorisation.

Table 2: Clinical review with pragmatic clinical categorisation of the 203 children flagged from questionnaire responses.

**Clinical sub-group amongst children with clinical concern for a post-COVID-19 condition (PCC)**	**n (N=203)**	**% (95% CI)**
Unable to contact*	34	
Reviewed but unable to be categorised	13	7·7 (4·2-12·8)
Recovered	63	37·3 (30·0 – 45·0)
Mental health issues	10	5·9 (2·9 - 10·6)
New acute respiratory tract infection^	3	1·8 (0·4 - 5·1)
Pre-existing condition (exacerbated by COVID-19)	18	10·1 (6·0 - 15·6)
Single organ dysfunction	38	22·5 (16·4 - 29·5)
- *Loss taste and/or smell*	*15*	
- *New wheezing*	*8*	
- *Nasal irritation/rhinitis*	*7*	
- *Diarrhoea*	*2*	
- *Tics/myoclonus*	*1*	
Persistent symptoms (multi-organ)#	10	5·3 (2·5 - 9·9)
Post-viral fatigue#	16	9·5 (5·5 - 14·9)
Long COVID compatible	*21*	*12·4 (7·9 - 18·4)*

*These cases excluded from the denominator (n=169) for calculations

^One child had a repeat SARS-CoV-2 infection.

#These sub-groups considered potentially compatible with UK consensus definition of Long COVID depending on functional impacts; 5 children lacked a significant impact on daily functioning.

Table 3: Demographics, symptoms, level of impairment amongst responder groups both flagged for clinical review and not.

	**Responders Not Flagged† (N=1278)**	**Flagged (Self-identified) Reviewed but no PCC (N=93)**	**Flagged (Self-identified) Reviewed and confirmed PCC (N=63)**	**RR^ (95%CI)**
**Sex female**	750 (49.1)	35 (37·6)	37 (58·7)	1.45 (0.9-2.4)
**Age group**				
under 5 years	543 (35.5)	28 (30·1)	11 (17·5)	Ref
5 to 11 years	644 (43.5)	44 (47·3)	22 (34·9)	1.66 (0.8-3.4)
12 to 15 years	304 (19.9)	21 (22·6)	28 (44·4)	4.25 (2.1-8.4)***
16 or older	6 (0.4)	0	1 (1·6)	7.19 (1.1-48.4)*
Missing	11 (0.7)	0	1 (1·6)	4.20 (0.6-30)
**Aboriginal or Torres Strait islander**	40 (2.6)	3 (3·2)	0	-
**1st Quintile IRSAD**	698 (54.7)	35 (37·6)	31 (49·2)	1·15 (0·7-1·9)
**Risk Category during acute admission (Purple or Blue)#**	45 (2.9)	3 (3·2)	3 (4·8)	1·61 (0·5-4·9)
**Medical comorbidity**	1424 (93.2)	45 (48·4)	28 (44·4)	2.10 (1.3-3.4)**

Abbreviations: PCC, post-COVID condition; IRSAD, Index of relative socio-economic advantage and disadvantage

†Data reported as n (%) in sub-category unless otherwise specified.

^Relative risk of variable comparing cases flagged, reviewed and confirmed PCC with responders not flagged for concern.

#Children were assigned to blue (moderate), or purple (high) risk categories due to high number of risk factors and/or met clinical criteria (e.g. high fever, decreased O2 saturation).

* p value <0.05, ** p value <0.005, *** p value <0.0001.
